# Non-reducible knee dislocation with interposition of the vastus medialis muscle

**DOI:** 10.1007/s10195-011-0134-2

**Published:** 2011-04-21

**Authors:** Alessandro Bistolfi, Giuseppe Massazza, Federica Rosso, Stefano Ventura, Enzo Cenna, Luca Drocco, Maurizio Crova

**Affiliations:** 1 Department of Orthopaedics and Traumatology, CTO/M Adelaide Hospital, Turin, Italy; 2 University of the Studies of Turin, Via Zuretti 29, 10126 Turin, Italy

**Keywords:** Knee dislocation, Muscle interposition, Irreducibility

## Abstract

Irreducibility of the knee following complete dislocation is a rare event determined by the interposition of various capsulo-ligamentous structures in the joint space. Such cases often require urgent surgical treatment. We report the case of a healthy 70-year-old man with a sprain of the left knee that occurred after a sports trauma. The patient showed knee dislocation with multiple ligamentous injuries and articular block due to interposition of a portion of the vastus medialis muscle. After arthroscopic evaluation, we performed surgical treatment to free the muscle, regularize the medial meniscus and suture the posterior and medial capsule and ligaments; the cruciate ligaments were not treated. The most interesting aspect of the articular damage in this case was a wide detachment of the vastus medialis muscle with intra-articular dislocation. The decision to treat only the posterior lesions and allow the healing of the front ones by rehabilitation treatment was supported by full functional recovery and return to sports activity.

## Introduction

Knee dislocation is a complex trauma often characterized by association of several capsulo-ligamentous lesions; its treatment remains controversial [1]. For rational planning and treatment, it is important to define exactly the degree of dislocation and the complexity of the associated lesions. Various scales have been proposed, based either on the position of the dislocation (defined from the relation between femur and tibia) or on the type of the associated ligamentous lesions [2]. Magnetic resonance imaging (MRI) seems to be the most appropriate radiological examination [3, 4]. Sometimes, clinical appraisal under anaesthesia can be useful [5]. Arteriography and electroneuromyography are indicated in case of suspicion of neuro-vascular lesions, which may need immediate surgical treatment [6].

Other cases of knee irreducibility determined by interposition of various capsulo-ligamentous structures have been previously described. Nevertheless, to our knowledge, there are very few reports of interposition of the vastus medialis muscle.

## Case report

G.R., a healthy, sporty (skiing), 70-year-old man, had a sprain of the left knee with a valgus-external spin trauma after a crash against another skier at low–medium speed. He reported immediate pain and loss of function at the knee and was immediately taken to the emergency room.

**Fig. 1:**
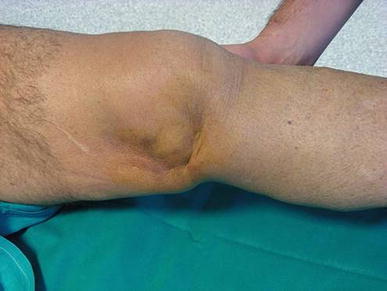
Skin pucker with recess at the level of the medial joint line

The clinical appraisal showed that the left knee was fixed in a valgus-flexed attitude, with pain on the medial compartment and a large skin depression on the medial side of the joint line (Fig. 1). Severe reduction of range of motion was reported (maximum flexion 60° and 30° of lack of extension). Valgus stress test, medial meniscus tests, Lachman test, front drawer and posterior drawer tests were all positive. Neuro-vascular lesions were absent.

**Fig. 2:**
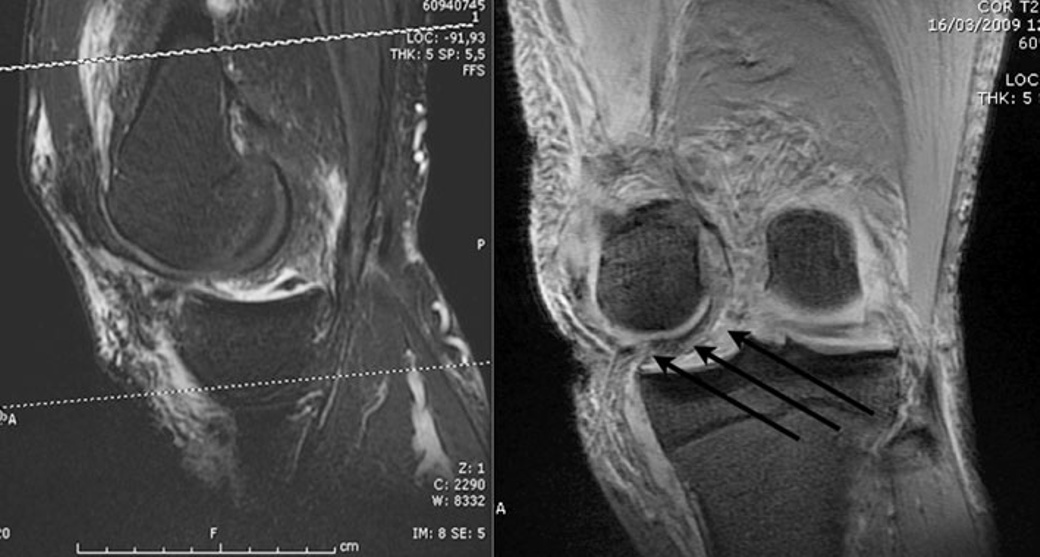
MRI showing muscular-density-like structure (indicated by*arrows*), which turned out to be intra-articularly dislocated

The radiographic evaluation showed a subluxation of the knee with abnormal wideness of the medial joint space. The MRI showed anterior subluxation of the medial femoral condyle with intra-articular dislocation of the anterior-medial soft tissues. It also showed: complete lesion of both anterior and posterior cruciate ligaments (ACL and PCL), alteration and fragmentation of the medial meniscus (MM), rupture of the medial collateral ligament (MCL) and, finally, lateral subluxation of the patella. The MRI also showed, in the medial side, a muscular-density-like structure, which turned out to be intra-articularly located and to enter in contact with the medial femoral condyle (Fig. 2).

Given the diagnosis of knee dislocation with multiple ligamentous injuries (ACL, PCL, MM, MCL) and muscle interposition, surgical treatment appeared to be necessary.

**Fig. 3:**
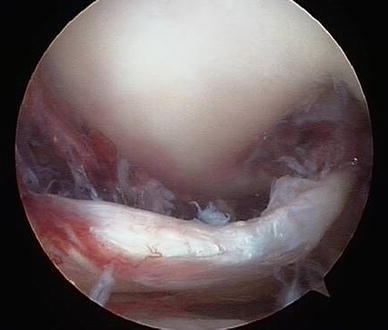
Arthroscopy showed the intra-articular dislocation of the vastus medialis

During the arthroscopy, a wide medial capsular lesion with intra-articular dislocation, a complex bucket-handle injury of MM and proximal disinsertion of PCL and ACL were observed. Specifically, dislocation of the vastus medialis was revealed (Fig. 3).

Because it was impossible to reduce the dislocation of the vastus medialis by arthroscopy, a medial access arthrotomy was performed. It was thus possible to appreciate the following lesions: a distal complete laceration of the MCL, of the popliteal oblique ligament (POL) and of the posterior-medial capsule and a complete laceration of the meniscus-femoral ligament. Moreover, there was a dislocation of the vastus medialis muscle, whose distal portion was dislocated into the intra-articular space through the capsular lesion.

**Fig. 4:**
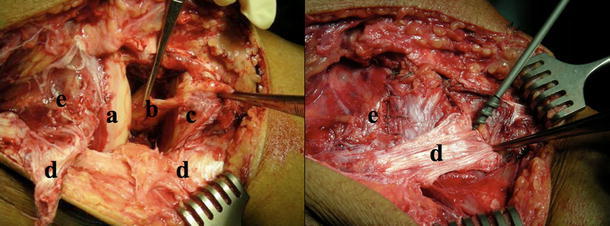
Intra-operative reconstruction of vastus medialis muscle and of the MCL:*a*, medial condyle;*b*, damaged medial meniscus, dislocated in the intercondylar notch;*c*, tibia;*d*, MCL;*e*, vastus medialis muscle

Firstly, the release of the muscle belly from the notch was performed. This restored the mobility and the alignment of the knee joint and gave a better view of the capsular and ligamentous lesions. Then, selective partial regularization of the MM was performed. Finally, plastic suture of the posterior-medial and medial capsule, re-insertion of the POL with detached points and suture of the MCL with small anchor type “T-corkscrew” were performed (Fig. 4).

After the surgery, the knee showed a correct alignment and the varus-valgus stress tests were negative. The rehabilitation program was immobilization with 20° of flexion for 15 days, then progressive recovery of range of motion in an articulated knee brace for 30 days. Partial weight-bearing was allowed after 15 days, and full weight-bearing after 45 days.

**Fig. 5:**
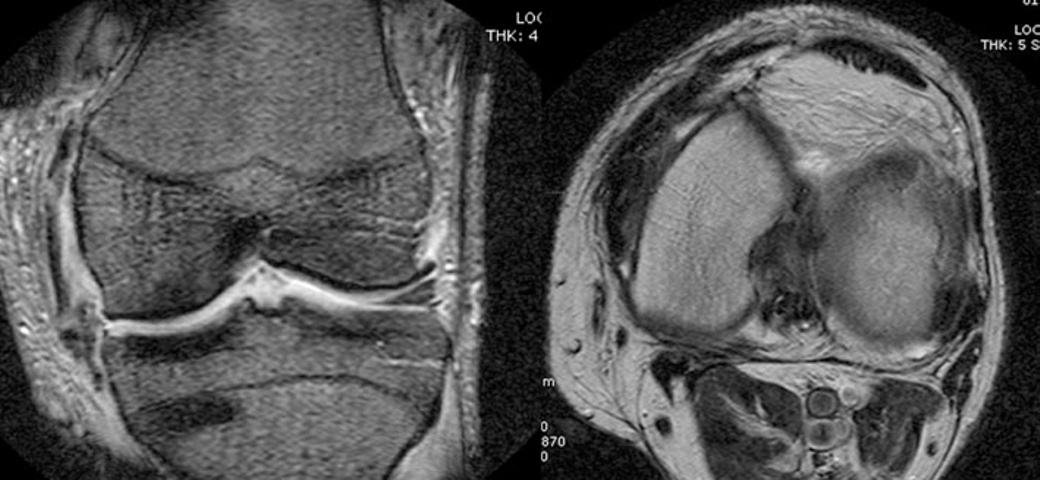
MRI at 6 months showing the healing of the MCL, with residual oedema

We evaluated the patient at 6 months after the trauma through a clinical and instrumental evaluation with MRI and ultra-sonographic examination (US). The MRI showed the rupture of ACL, an altered morphology of PCL and a thickness increase with residual oedema of the MCL that appeared completely re-inserted (Fig. 5). To obtain a more detailed evaluation of the muscular situation, US was performed; it confirmed the thickness increase in MCL and showed continuity of vastus medialis with minimal oedema. It also showed no significant differences between right and left knee regarding the morphology of the vastus medialis muscle.

The clinical examination showed a complete range of motion (full extension, flexion of 130°) with no lateral instability; Lachman and posterior drawer tests were minimal positive. This minimal instability was completely asymptomatic in the patient’s daily life. The quadriceps fully recovered after the rehabilitation. The patient walked, hiked, cycled and ran after 6 months and returned to alpine skiing after 10 months, as an active, healthy, 70-year-old man can do.

During the most recent clinical examination, the patient gave his consent for publication of this case report.

## Discussion

The interesting aspect of these types of knee dislocations is a wide lesion of the medial capsulo-ligamentous structures and their interposition between the femoral condyles, which causes the irreducibility of the dislocation itself. It is usually the result of a sprain in posterior-lateral sense. The presence of a cutaneous retraction on the medial joint line and eventual signs of cutaneous suffering are characteristic clinical signs [7, 8].

The few cases described in the literature generally refer to the interposition of various medial capsulo-ligamentous structures, and their authors propose diverse surgical solutions without general agreement, particularly concerning the repair of ACL and PCL [9].

The particularity of our case is a wide detachment of the vastus medialis muscle with intra-articular dislocation. This lesion clinically corresponded to an articular block with a forced valgus deviation of the limb and, specifically, to a cutaneous retraction at the level of the joint line. The intra-articular muscular interposition was detected by MRI and confirmed by the arthroscopy.

The first arthroscopic step is advised in the literature, because it allows direct appraisal of the lesions and their eventual repair [10], but generally, in case of multiple complex lesions such as that described herein, arthrotomy is necessary [1, 11, 12]. Concerns remain regarding the treatment of the capsulo-ligamentous lesions.

To our knowledge, very few cases presenting a similar condition can be found in the literature. Kilicoglu et al. in 2001 reported the case of a patient with an irreducible knee dislocation with interposition between the femoral condyles of the vastus medialis muscle. The clinical appraisal and the radiologic findings were similar to our case. In contrast to our approach, their surgical choice was to section the muscle to allow reduction of the knee dislocation and to perform the reconstruction of the ACL [13]. Silverberg et al. published a similar case in 2004. In this case the surgical choice was to remove the border of the vastus medialis, to reduce the dislocation and to insert the MCL through small anchors. In that case, a PCL repair was performed [14].

The difference in our case is that we chose to repair the rupture of the vastus medialis muscle and to relocate it in its anatomical site. This procedure allowed us to reduce the knee dislocation and to repair the medial capsulo-ligamentous lesions. In addition, in our case a lesion of the popliteus oblique ligament (POL) was also present and was repaired, in accordance with the literature [11]. Another difference is that we decided not to treat the ACL or PCL. This decision was taken in accordance with the literature [12] and in consideration of the age of the patient, as well as his functional demands. After the first 45 days, the patient underwent the same rehabilitation protocol that we apply after arthroscopic reconstruction of the ACL. This choice was supported by the results: immediate lateral stability, full functional recovery and complete return to sports activity as before the trauma.

In the end, what we have found in our experience is that, in this kind of lesion, lateral and rotational stability must be achieved immediately with reconstruction or suture of the ligaments, while reconstruction of the cruciate ligaments can be postponed and even avoided in elderly patients or those not engaged professionally in sports. In this case the rehabilitation protocol and the stability provided by the reinforcement of the muscles play a fundamental role.
